# Pathophysiological importance of bile cholesterol reabsorption: hepatic NPC1L1-exacerbated steatosis and decreasing VLDL-TG secretion in mice fed a high-fat diet

**DOI:** 10.1186/s12944-019-1179-0

**Published:** 2019-12-28

**Authors:** Yu Toyoda, Tappei Takada, Yoshihide Yamanashi, Hiroshi Suzuki

**Affiliations:** Department of Pharmacy, The University of Tokyo Hospital, Faculty of Medicine, The University of Tokyo, 7-3-1 Hongo, Bunkyo-ku, Tokyo, 113-8655 Japan

**Keywords:** Cholesterol transporter, Ezetimibe, Hepatic stress, Lipotoxicity, Reabsorption of biliary secreted cholesterol, Steatosis model animal

## Abstract

**Background:**

Non-alcoholic fatty liver disease (NAFLD) is one of the most common liver diseases worldwide, although its pathogenesis remains to be elucidated. A recent study revealed that hepatic Niemann-Pick C1-Like 1 (NPC1L1), a cholesterol re-absorber from bile to the liver expressed on the bile canalicular membrane, is an exacerbation factor of NAFLD. Indeed, transgenic mice with hepatic expression of human NPC1L1 under a liver-specific promoter (L1-Tg mice) developed steatosis with a high-fat diet (HFD) containing cholesterol within a few weeks. However, the mechanism underlying diet-induced hepatic NPC1L1-mediated lipid accumulation is poorly defined.

**Methods:**

To achieve a deeper understanding of steatosis development in L1-Tg mice, the biochemical features of hepatic NPC1L1-mediated steatosis were investigated. Hemizygous L1-Tg mice and wild-type littermate controls fed a HFD or control-fat diet were used. At the indicated time points, the livers were evaluated for cholesterol and triglyceride (TG) contents as well as mRNA levels of hepatic genes involved in the maintenance of lipid homeostasis. The hepatic ability to secrete very low-density lipoprotein (VLDL)-TG was also investigated.

**Results:**

Unlike the livers of wild-type mice that have little expression of hepatic Npc1l1, the livers of L1-Tg mice displayed time-dependent changes that indicated steatosis formation. In steatosis, there were three different stages of development: mild accumulation of hepatic cholesterol and TG (early stage), acceleration of hepatic TG accumulation (middle stage), and further accumulation of hepatic cholesterol (late stage). In the early stage, between WT and L1-Tg mice fed a HFD for 2 weeks, there were no significant differences in the hepatic expression of Pparα, Acox1, Fat/Cd36, Srebf1, and Srebf2; however, the hepatic ability to secrete VLDL-TG decreased in L1-Tg mice (*P* < 0.05). Furthermore, this decrease was completely prevented by administration of ezetimibe, an NPC1L1-selective inhibitor.

**Conclusion:**

Hepatic NPC1L1 exacerbates diet-induced steatosis, which was accompanied by decreased hepatic ability of VLDL-TG secretion. The obtained results provide a deeper understanding of L1-Tg mice as a promising NAFLD animal model that is able to re-absorb biliary-secreted cholesterol similar to humans. Furthermore, this work supports further studies of the pathophysiological impact of re-absorbed biliary cholesterol on the regulation of hepatic lipid homeostasis.

## Background

Non-alcoholic fatty liver disease (NAFLD) is a major cause of liver diseases characterized by the presence of hepatic steatosis and of increasing serious concern to human health; its prevalence has been estimated to be approximately 25% of the global population [1, 2]. Despite substantial progress in current basic and clinical investigation for NAFLD, its pathogenesis remains to be elucidated and no agent is approved yet for this multifactorial abnormal liver condition [3]. Recent findings suggest a link between hepatic cholesterol accumulation and NAFLD progression, although the mechanisms by which hepatic cholesterol promotes NAFLD development are not well understood [4–6]. In humans, hepatic cholesterol is derived not only from de novo synthesis and its circulation, but also from bile (reuptake of biliary secreted cholesterol), which is mediated by Niemann-Pick C1-Like 1 (NPC1L1), a cholesterol re-absorber expressed on the bile canalicular membrane [7]. Thus, how hepatic NPC1L1 is involved in the pathogenesis of NAFLD should be of great interest.

NPC1L1 is a cholesterol transporter that was identified as the molecular target of ezetimibe, a globally used dyslipidemia agent that inhibits intestinal cholesterol absorption [8]. In humans, NPC1L1 is highly expressed in both the intestine and liver; however, in rodents, *Npc1l1* is predominantly expressed in the intestine, not the liver. Hitherto, in vivo studies of NPC1L1 expression in the livers of mice using adenovirus-mediated or germ-line transgenics have shown that hepatic NPC1L1 negatively regulates biliary cholesterol excretion [7, 9, 10]. These results suggest that hepatic NPC1L1 could mediate reabsorption of cholesterol in bile. Indeed, hepatic NPC1L1 re-absorbs cholesterol from bile. Nevertheless, due to the species differences in NPC1L1 tissue distribution, the pathophysiological impact of hepatic NPC1L1 on liver diseases has been overlooked in a lot of previous studies using murine models. Regarding this issue, using transgenic mice with hepatic expression of human NPC1L1 under a liver-specific ApoE promoter (L1-Tg mice) [7], a recent study identified hepatic NPC1L1 as an NAFLD risk factor amendable to therapeutic intervention [11]. Indeed, L1-Tg mice fed with a western diet exhibited steatosis characterized by the elevation of hepatic cholesterol and triglyceride (TG) levels within a few weeks, which was prevented and rescued by the administration of ezetimibe. Considering that the expression levels of hepatic NPC1L1 in L1-Tg mice are relatively similar to that of humans [7], L1-Tg mice with diet-induced steatosis are expected to be a useful model for investigating the developmental mechanisms of NAFLD and exploring new therapeutic targets. However, the mechanism underlying hepatic NPC1L1-mediated lipid accumulation in the liver remains poorly defined.

In this study, we investigated the biochemical features of hepatic NPC1L1-mediated steatosis to aid further understanding of NAFLD development in L1-Tg mice. The data implied the pathophysiological importance of re-absorbed biliary cholesterol in the regulation of hepatic lipid homeostasis.

## Methods

### Materials

The following compounds were purchased from the indicated commercial sources: ezetimibe (Sequoia Research Products, Pangbourne, UK), tyloxapol (Sigma Aldrich, St. Louis, MO, USA). All other chemicals used were commercially available and were of analytical grade.

### Animals

Transgenic mice expressing human NPC1L1 in hepatocytes (L1-Tg mice) [7] (B6;D2-Tg(APOE-NPC1L1)20Lqyu/J) were purchased from The Jackson Laboratory (Bar Harbor, Maine, USA) and backcrossed at least eight generations to C57BL/6 J mice (Japan SLC, Shizuoka, Japan) as described previously [11]. All experiments used hemizygous L1-Tg mice and WT littermate controls. The mice used in this study were males that were 6–12 weeks of age and were maintained on a standard diet and water ad libitum under a 12 h/12 h light/dark cycle that started at 7:00. As a control-fat diet (CFD) and high-fat diet (HFD) for mice, CLEA Rodent Diet CE-2 (CLEA Japan, Tokyo, Japan) and D15002 (CE-2 with 1% cholesterol, 0.5% cholic acid, and 10% palm oil; CLEA Japan) were used, respectively.

Male mice from each litter were weaned and genotyped at 4 w and then fed a CFD for up to 6 w of age, when the dietary administration was started in each randomly assigned group of mice. Diets containing ezetimibe (16 μg/g diet) were made by mixing powdered HFD with ezetimibe before use. Of note, it was previously confirmed that the dose of ezetimibe used in the present study is enough for chemical inhibition of hepatic NPC1L1 in the liver of mice fed the HFD with ezetimibe [11]. At the indicated time points, blood specimens were taken immediately and serum specimens were prepared as described previously [11]. Bile specimens from each mouse were collected by cannulation under the deep anesthesia with urethane [1.25 g/kg body weight (B.W.), intraperitoneal administration] (Sigma Aldrich) as described previously [12]. In brief, the bile duct was cannulated with a Teflon-coated tube (UT-03 type) (Unique medical, Tokyo, Japan). Collected bile specimens were weighed, and bile volume was determined by assuming a specific gravity of 1.0 g/mL. At necropsy, livers and epididymal adipose tissues (EATs) were excised and weighed, and the livers were rapidly frozen and stored in liquid nitrogen until further processing. Other specimens were stored at − 80 °C until use.

### Immunoblotting

Preparation of liver lysate samples and immunoblotting were conducted as described previously [11]. Briefly, frozen livers were weighed and defrosted on ice, then homogenized (g of tissue/20 mL) using an ice-cold Physcotron homogenizer (Microtec, Chiba, Japan) in ice-cold RIPA lysis buffer containing a Protease Inhibitor Cocktail for General Use (Nacalai Tesque, Kyoto, Japan). Crude lysates were incubated at 4 °C for 30 min with gentle rotation, then subjected to centrifugation at 20,000×*g* at 4 °C for 30 min. The resulting supernatant was carefully collected in a new tube, and the protein concentration was determined using the BCA Protein Assay Kit (Pierce, Rockford, IL, USA) with BSA as a standard according to the manufacturer’s protocol.

Subsequently, the liver lysate samples were separated by sodium dodecyl sulfate polyacrylamide gel electrophoresis and transferred to an Immobilon-P PVDF membrane (Millipore, Bedford, MA, USA) by electroblotting at 15 V for 51 min. For blocking, the membrane was incubated in Tris-buffered saline containing 0.05% Tween 20 and 3% BSA (TBST-3%BSA). Blots were probed with a Rabbit polyclonal anti-NPC1L1 (Novus Biologicals, Littleton, CO, USA; Cat# NB400–128; 1:1000 dilution in TBST-0.1%BSA) and a Donkey anti-rabbit IgG-horseradish peroxidase (HRP)-conjugate (GE Healthcare, Buckinghamshire, UK; Cat# NA934V; 1:2000 dilution in TBST-0.1%BSA) for 1 h at room temperature, respectively. After washing in TBST for 1 h at room temperature, HRP-dependent luminescence was developed with ECL™ Prime Western Blotting Detection Reagent (GE Healthcare) and detected using a luminescent image analyzer (Bio-Rad Laboratories, Tokyo, Japan).

### Lipid extraction

Lipid extraction from liver samples was performed according to the Bligh and Dyer method as described previously [11]. For quantitative calibration curves, standard samples containing known concentrations of cholesterol and TG were prepared in a similar manner to the experimental samples.

### Biochemical measurements

The concentrations of cholesterol, bile acids, phospholipids and TG, and the activity of alanine aminotransferase (ALT) in each sample were measured using commercially available kits according to manufacturer’s instructions. The Cholesterol E-test Wako Kit, the TBA test Wako Kit (for the determination of total bile acids), the Phospholipids C-test Wako Kit, the Triglyceride E-test Wako Kit, and the Transaminase CII-test Wako Kit (Wako Pure Chemical Industries, Tokyo, Japan) were used in this study.

### Determination of hepatic VLDL-TG-secretion rate

To quantify hepatic very low-density lipoprotein-triglyceride (VLDL-TG) secretion, the rate of TG accumulation in the blood was measured as described previously [13] with some modifications. Briefly, blood was drawn from the neck vein of fasted mice for baseline measurements and then tyloxapol (100 mg/mL) in saline (Otsuka Pharmaceutical, Tokyo, Japan) was intravenously administered (500 mg/kg of B.W.] to block the peripheral removal of newly secreted VLDL. Three hours after administration, blood was collected and then subjected to TG measurement. Linearity in the time-dependent increase in the blood TG levels during the period had been confirmed previously [14]. The hepatic VLDL-TG-secretion rate [mg of TG/dL of serum/h] was calculated as a slope of the TG concentration vs. time.

The liver’s apparent VLDL-TG secretion ability into the blood [g of liver/dL of serum/h] was calculated by dividing the VLDL-TG secretion rate [mg of TG/dL of serum/h] by the average hepatic TG levels [mg of TG/g of liver]. Uncertainty in the quotients were calculated according to a general formula for error propagation. Since the tyloxapol treatment for 3 hours could affect hepatic TG levels at necropsy, an average value of hepatic TG levels in each experimental group that was separately measured using untreated mice was used. Some of the average values were derived from the previous study [11].

### RNA extraction and qRT-PCR

Total RNA was extracted from mouse livers using the RNA isoPlus® Reagent (Takara, Shiga, Japan), following the manufacturer’s protocol. Reverse transcriptional reaction and subsequent qRT-PCR using SYBR® GreenER™ qPCR SuperMix Universal (Life Technologies, Tokyo, Japan) were performed as described previously [11]. The expression levels of each gene were normalized to that of β-actin. The sequences of the primers used are shown in Additional file 1: Table S1.

### Statistical analyses

All statistical analyses were performed by using Excel 2013 (Microsoft Corp., Redmond, WA, USA) with Statcel4 add-in software (OMS publishing, Saitama, Japan). Different statistical tests were used for different experiments as described in the figure legends which include the numbers of biological replicates (*n*). Briefly, when analyzing multiple groups, the similarity of variance between groups was compared using Bartlett’s test. When passing the test for homogeneity of variance, a Dunnett’s test for comparisons with a control group or a parametric Tukey–Kramer multiple-comparison test for all pairwise comparisons was used; otherwise a non-parametric Shirley–Williams’s multiple-comparison test for trend analysis or a non-parametric Steel–Dwass test for multiple comparisons was used. With single pairs of quantitative data, after comparing the variances of a set of data by *F*-test, unpaired Student’s or Welch’s *t*-test was performed. Statistical significance was defined in terms of *P* values less than 0.05 or 0.01.

## Results

### Hepatic NPC1L1-mediated decrease in biliary cholesterol secretion in mice fed a HFD

**Fig. 1:**
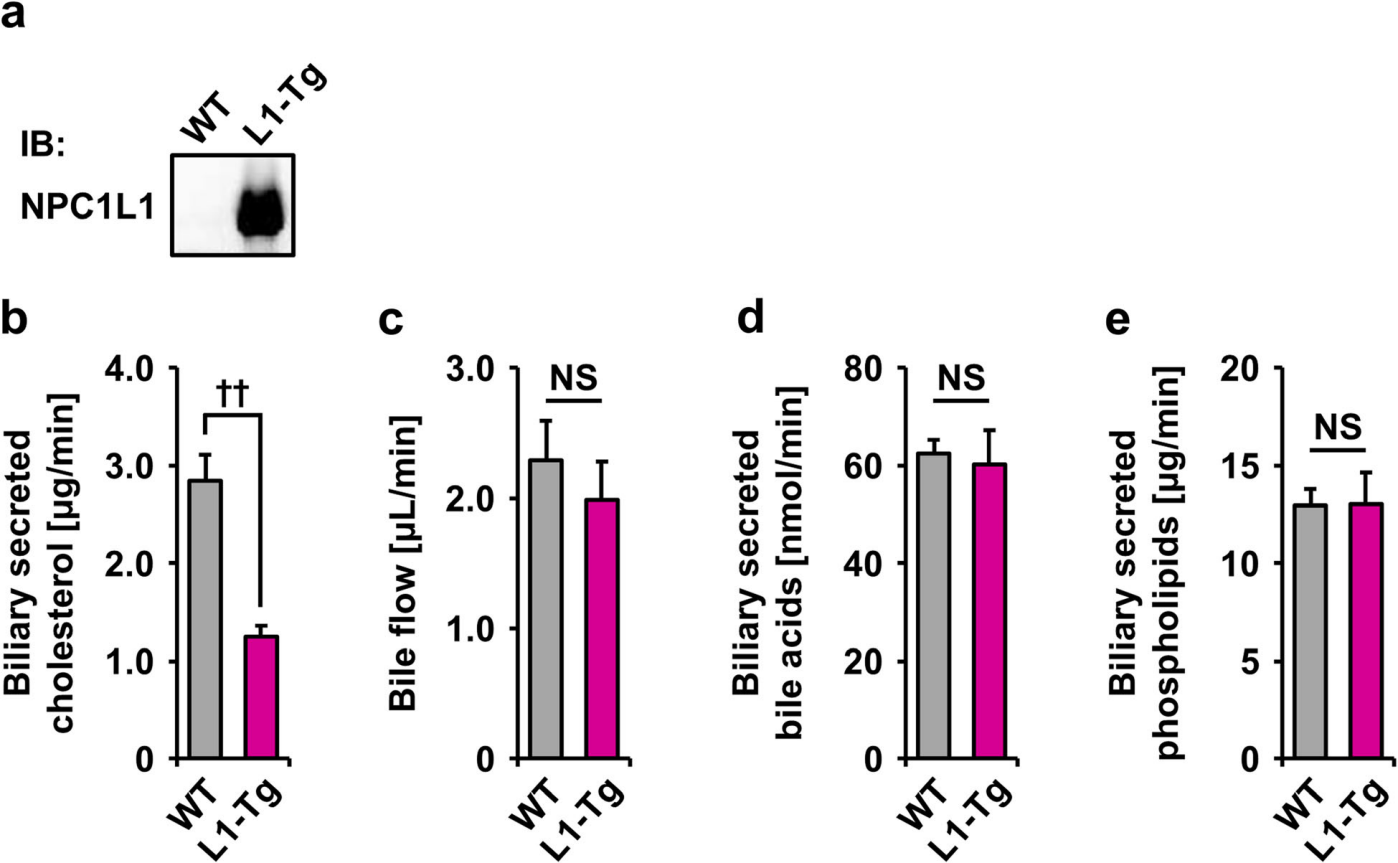
Decrease in the biliary cholesterol secretion in L1-Tg mice. **a** Immunoblot detection of hepatic NPC1L1 protein in L1-Tg mice using an anti-NPC1L1 antibody. WT, wild-type. **b** Lower rates of biliary cholesterol secretion in L1-Tg mice fed a high-fat diet (HFD) for 2 weeks than those in WT mice. **c** Bile flow. **d** Biliary secretion of bile acids. **e** Biliary secretion of phospholipids. Data are expressed as the mean ± SEM. *n* = 4 (WT), 6 (L1-Tg). Statistical analyses for significant differences were performed using a two-sided *t*-test (††, *P* < 0.01; NS, not significantly different between groups)

Hepatic expression of NPC1L1 in L1-Tg mice was verified by immunoblotting using the anti-NPC1L1 antibody (Fig. 1a). To confirm that the hepatic NPC1L1 could mediate reabsorption of biliary cholesterol into the liver of L1-Tg mice under HFD feeding, biliary secretion of cholesterol was investigated in each type of mice fed a HFD for 2 w (Fig. 1b), an enough period for steatosis formation in L1-Tg mice. As expected, net secretion rates of cholesterol into bile of L1-Tg mice were significantly lower than those of WT mice, which is consistent with previous studies showing the reduction in biliary cholesterol in L1-Tg mice fed a non-high-fat diet [7, 11]. At this time, bile flow (Fig. 1c); biliary secretion of bile acids (Fig. 1d) and phospholipids (Fig. 1e) were almost comparable between the WT and L1-Tg mice.

With *ATP-binding cassette transporter g5* (*Abcg5*) and *Abcg8*—physiologically important biliary cholesterol exporters [15], there were little differences in their mRNA levels between the liver of WT and L1-Tg mice fed a HFD (Additional file 1: Figure S1), indicating that the reduction in biliary cholesterol secretion in the L1-Tg mice was not coupled to decreased hepatic expression of these major cholesterol transporters from the liver to bile. Additionally, no appreciable changes were detected in the hepatic mRNA levels of *Abcb11* (encoding an exporter of bile acids into bile) and *Abcb4* (encoding a critical requirement for biliary secretion of phospholipids) (Additional file 1: Figure S1) of which conventional knockout in mice reportedly resulted in the considerable increase [16] and decrease [17] of biliary cholesterol secretion, respectively.

### Hepatic NPC1L1-mediated exacerbation of steatosis in mice

**Fig. 2:**
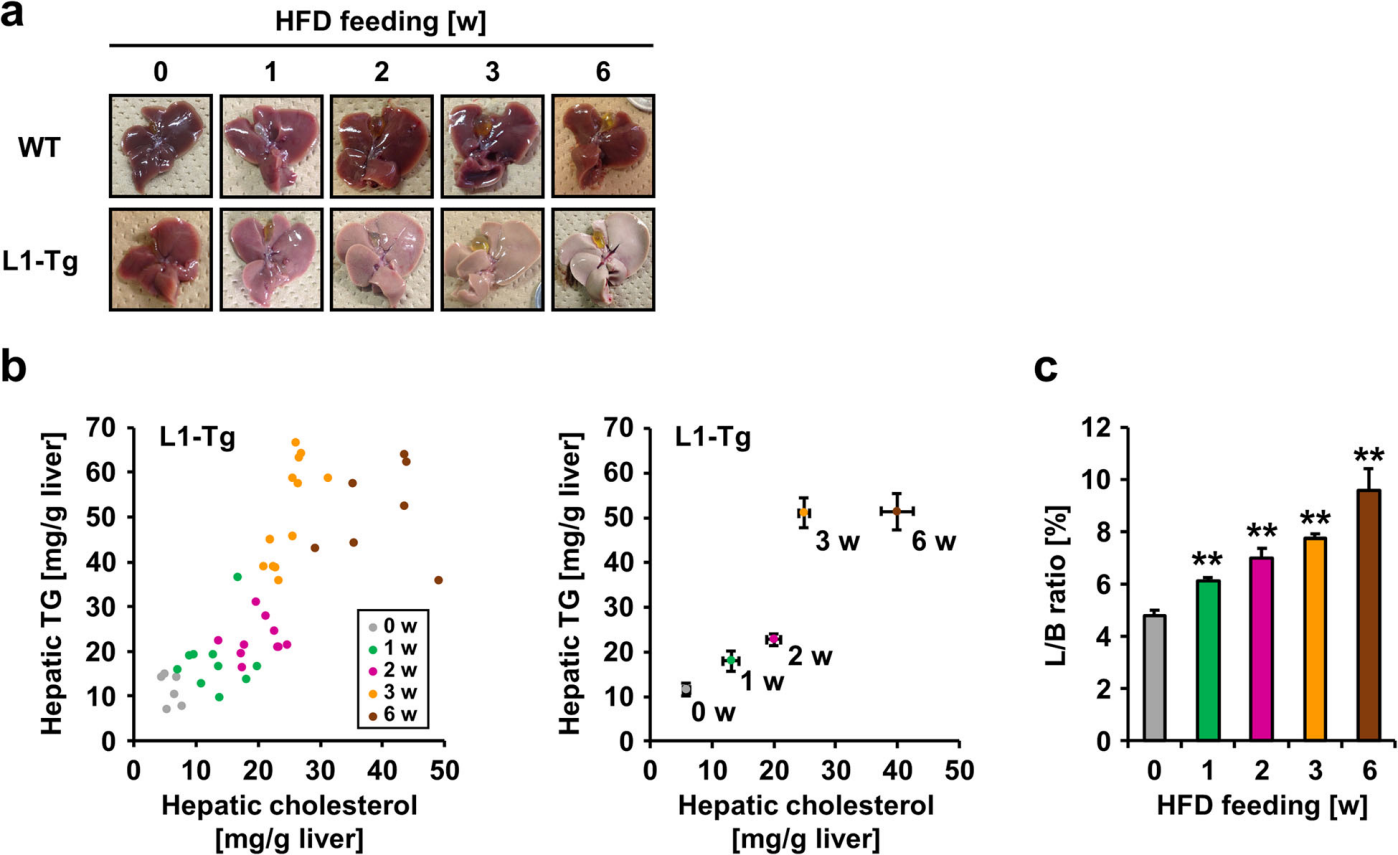
Time-dependent progression of hepatic NPC1L1-mediated steatosis in L1-Tg mice**. a** Photographic images of livers from wild-type (WT) and L1-Tg mice fed a high-fat diet (HFD) at independent period. **b** Time-dependent changes in hepatic levels of cholesterol and triglyceride (TG). To visualize association between cholesterol and TG levels in individual mouse livers, a scatter diagram showing hepatic cholesterol levels on the X axis and TG levels on the Y axis was created for each group of L1-Tg mice fed a HFD for the indicated time periods (w, weeks). All data in the *left* panel were summarized in the *right* panel as the mean ± SEM; where bars are not shown, the SEM is within the limits of the closed symbols. The data from the 6 w group were derived from our previous study [11] under a CC-BY license. **c** Time-dependent changes in the ratios of liver weight to body weight (L/B ratios) in L1-Tg mice fed a HFD for the indicated number of weeks. Data are expressed as the mean ± SEM. *n* = 6, 8, 6, 12, 7 (0, 1, 2, 3, 6 w). Statistical analyses for significant differences were performed using Bartlett’s test, followed by a non-parametric Shirley–Williams’s multiple-comparison test (**, *P* < 0.01 vs. 0 w control). The values of mean and SEM are summarized in Additional file 1: Appendix

To address the biochemical features of steatosis caused by hepatic NPC1L1, feeding period-dependent lipid accumulation in the livers of L1-Tg mice fed a HFD was explored (Fig. 2). Unlike the livers of WT mice, those of L1-Tg mice displayed remarkable time-dependent changes that suggested the steatosis formation (Fig. 2a). Individual levels of hepatic cholesterol and TG in L1-Tg mice fed a HFD for each experimental period are summarized as a scatter plot in Fig. 2b. Both hepatic cholesterol and TG levels in L1-Tg mice fed a HFD increased gradually during the experimental period (0–3 w) (Fig. 2b) and was accompanied by an increase in the ratios of liver weight to body weight (L/B ratios) (Fig. 2c). These results are consistent with the previous study demonstrating that hepatic NPC1L1 is a steatosis exacerbation factor in L1-Tg mice continuously fed the same HFD for several weeks [11]. Based on these results, it is possible that there are three different stages during steatosis formation that the present study characterized as mild accumulation of hepatic cholesterol and TG (early stage: before 2 w), acceleration of hepatic TG accumulation (middle stage: 2–3 w), and further accumulation of hepatic cholesterol (late stage: after 3 w).

**Fig. 3:**
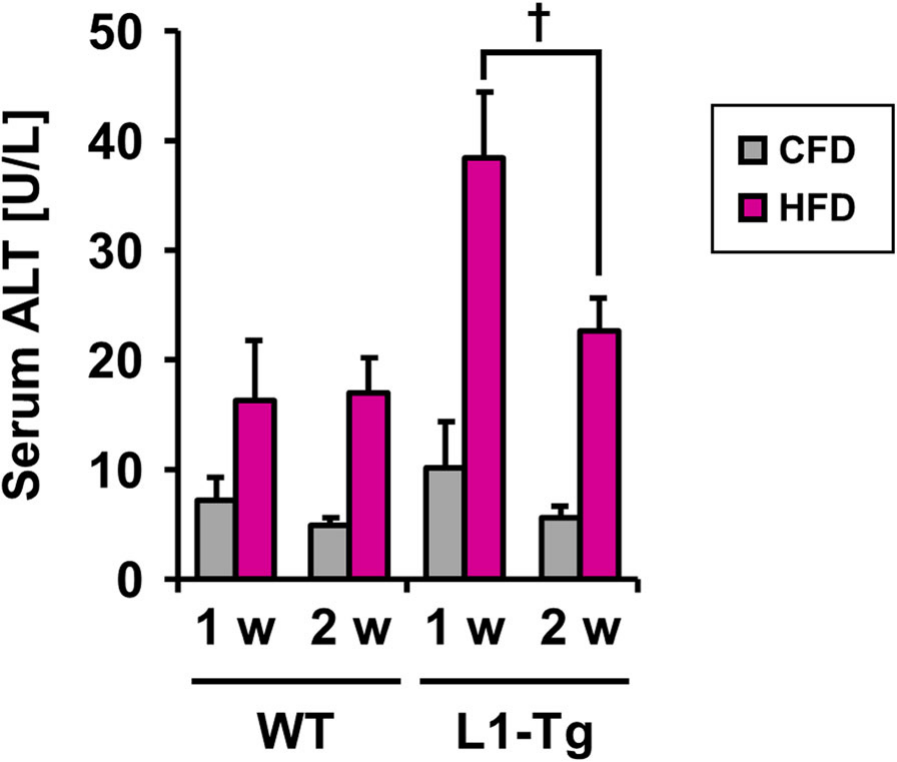
Serum ALT levels in WT and L1-Tg mice**.** Wile-type (WT) and L1-Tg mice were fed a control-fat diet (CFD) or high-fat diet (HFD) for the indicated periods, then serum ALT levels were determined as an indicator of liver injury. Data are expressed as the mean ± SEM. *n* = 7 (CFD), 4 (HFD) [1 w]; 4 (CFD), 7 (HFD) [2 w] in WT mice: 4 (CFD), 7 (HFD) [1 w]; 6 (CFD), 7 (HFD) [2 w] in L1-Tg mice. Statistical analyses for significant differences were performed using a two-sided *t*-test (†, *P* < 0.05 between groups)

During the early stage, the increase in hepatic cholesterol seemed to gradually stimulate hepatic TG accumulation. This interpretation agreed with the model that hepatic NPC1L1 should be a cholesterol re-absorber from bile and the prevention of hepatic NPC1L1-mediated steatosis formation by the administration of ezetimibe, an NPC1L1 inhibitor (Additional file 1: Figure S2). Additionally, at 1 w in the early stage, L1-Tg mice fed a HFD exhibited higher serum ALT levels than other groups (Fig. 3), which was moderately attenuated during synchronization with steatosis progression (1–2 w). As a related finding, the steatotic livers of L1-Tg mice fed a HFD exhibited higher mRNA levels of *lipocalin 2* (*Lcn2*) than the other groups (Additional file 1: Figure S3); *Lcn2* is reportedly up-regulated during inflammation and in response to cellular stress to evoke protective effects against liver injury [18].

### Decreased VLDL-TG secretion ability in steatotic livers of L1-Tg mice

**Fig. 4:**
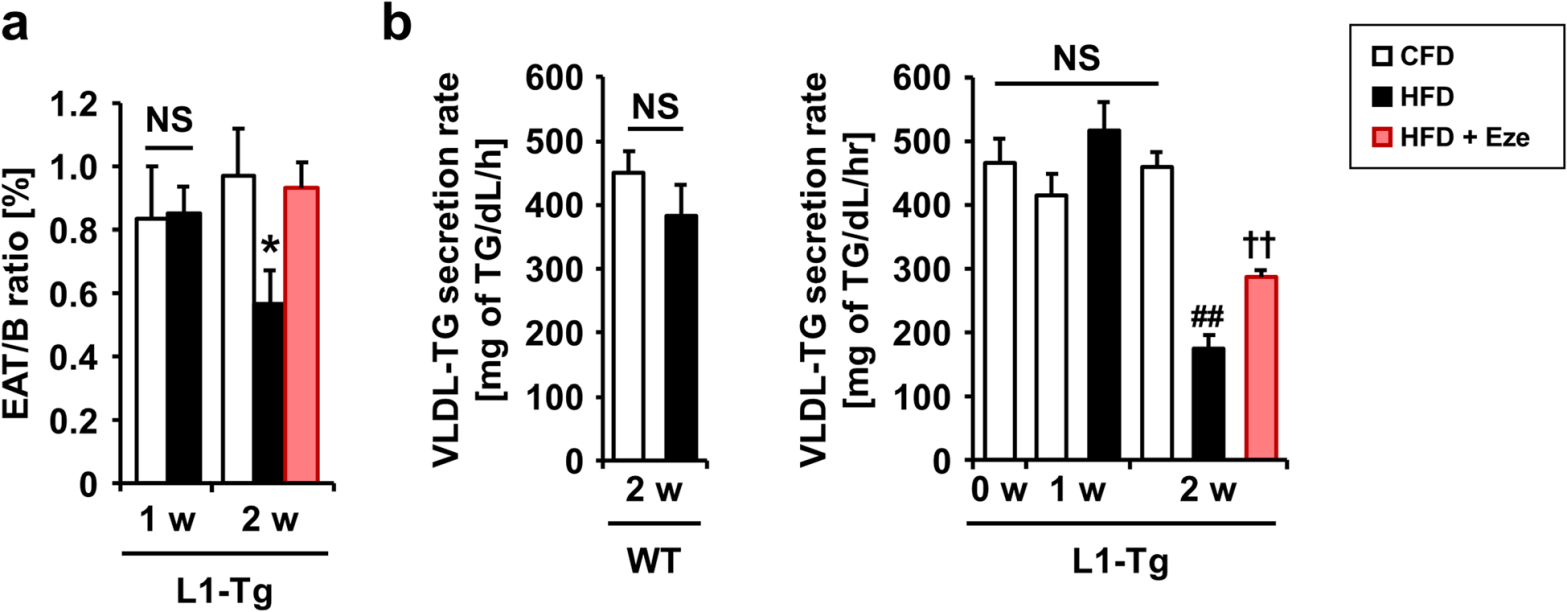
Biochemical characterization of hepatic NPC1L1-mediated steatosis in L1-Tg mice**. a** The ratios of epididymis adipose tissue weight to body weight (EAT/B ratios) in L1-Tg mice fed a control-fat diet (CFD) or high-fat diet (HFD) in the absence or presence of ezetimibe (Eze). w, weeks. *n* = 4 (CFD), 7 (HFD) [1 w]; 6 (CFD), 8 (HFD), 4 (HFD + Eze) [2 w]. **b** Effect of HFD feeding on very low-density lipoprotein-triglyceride (VLDL-TG)-secretion rates. As an index of the lipid-efflux rate from the liver to blood, we determined VLDL-TG-secretion rates in WT (*left*) and L1-Tg mice (*right*). *n* = 4 (CFD), 7 (HFD) [2 w] in WT mice: 4 (CFD) [0 w]; 4 (CFD), 7 (HFD) [1 w]; 6 (CFD), 7 (HFD), 4 (HFD + Eze) [2 w] in L1-Tg mice. Data are expressed as the mean ± SEM. Statistical analyses of significant differences among the groups in each category were performed using a two-sided *t*-test [NS, not significantly different between groups in (*a*) and (*b, left*); ††, *P* < 0.01 vs. 2 w-HFD group in (*b, right*)] or Bartlett’s test, followed by a Dunnett’s test [*, *P* < 0.05 vs. 2 w-CFD group as a control in (*a*; all 2 w groups)] or a parametric Tukey–Kramer multiple-comparison test [##, *P* < 0.01; NS, not significantly different among groups in (*b, right*; all non-ezetimibe groups)]

We further explored biological responses in the L1-Tg mice potentially linked to steatosis formation. Interestingly, the ratios of EAT weight to body weight (EAT/B ratios) in L1-Tg mice fed a HFD were lower than those fed a CFD at 2 w, despite the lack of a significant difference at 1 w (Fig. 4a). This decrease in the EAT/B ratios was prevented by ezetimibe administration. Considering the contrasting increase in the L/B ratios in L1-Tg mice with steatosis (Fig. 2c), it was hypothesized that the decrease in the amount of adipose tissue might reflect decreased lipid secretion from the liver. In fact, the liver is a major TG factory in the body that releases TG into the blood as a component of VLDL, which is a major secretion route of hepatic lipids. Therefore, hepatic VLDL-TG secretion was examined as described below. In addition, between WT and L1-Tg mice fed a HFD for 2 w, there were no significant differences in the hepatic mRNA levels of *Pparα*, *Acox1*, *Fat/Cd36*, *Srebf1*, and *Srebf2* (Additional file 1: Figure S4), all of which help maintain hepatic lipid homeostasis via processes such as lipid oxidation, fatty acid import, and lipogenesis. These results also suggest that changes in the VLDL-TG secretion (i.e. the other pathway governing hepatic lipid homeostasis) could be involved in steatosis formation in L1-Tg mice.

To examine whether VLDL-TG secretion from the liver decreased during hepatic NPC1L1-mediated steatosis formation, the secretion rate of lipids from the liver to the blood was investigated. For this purpose, a hepatic VLDL-TG-production assay following administration of tyloxapol (a lipoprotein lipase inhibitor) was conducted (Fig. 4b). While there was little difference in VLDL-TG-secretion rates between WT mice fed a CFD and HFD, those in L1-Tg mice fed a HFD for 2 w were significantly lower compared with the other groups. Moreover, this phenotype was attenuated by ezetimibe administration (Fig. 4b). For further evaluation, the apparent VLDL-TG-secretion ability from the liver to the blood (VLDL-TG-secretion rate / hepatic TG level) was calculated (Table 1). There was little difference in the VLDL-TG-secretion ability between WT mice fed with a CFD and HFD (Additional file 1: Figure S5); however, the values in the L1-Tg mice fed a HFD decreased in a feeding period-dependent manner (Table 1). This decrease was completely prevented by 2 w of ezetimibe administration. Moreover, serum TG levels in L1-Tg mice fed a HFD for 2 w were significantly lower than those after 1 w, which was rescued by ezetimibe (Additional file 1: Table S2).

**Table 1:** Apparent VLDL-TG-secretion abilities in the livers of L1-Tg mice fed a high-fat diet

Feeding time	Ezetimibe	Apparent VLDL secretion ability [g liver/dL/h]	*n*
0 w	–	40.5 ± 6.1^a^	4
1 w	–	28.8 ± 4.4^b^	7
2 w	–	6.5 ± 0.8^c^	7
2 w	+	39.1 ± 6.1^a^	4

Values are expressed as the mean ± SEM. Statistical analyses of significant differences among all the groups were performed using Bartlett’s test, followed by a non-parametric Steel–Dwass test for multiple comparisons. Different letters indicate significant differences between groups (*P* < 0.05); there were no significant differences between the groups indicated by same letters. Time-dependent decrease of apparent VLDL-TG-secretion ability was prevented by ezetimibe administration. VLDL-TG, very low-density lipoprotein-triglyceride; w, weeks. Details are shown in Additional file 1: Figure S5 with a bar chart of this information

We further addressed the mRNA levels of hepatic genes implicated in VLDL assembly and secretion (Additional file 1: Figure S6). These processes require both the synthesis of apolipoprotein B and the activity of microsomal triglyceride transfer protein encoded by the *ApoB* and *Mttp* genes, respectively [19]. In the mice treatment groups examined, mRNA levels of *ApoB* were not significantly different; however, the livers of L1-Tg mice fed a HFD for 2 w had lower mRNA levels of *Mttp* than the other groups, although the difference might not be large enough to plausibly explain the hepatic phenotype alone. Interestingly, a more drastic decrease was observed in the mRNA levels of ApoC3 of which translational product reportedly favors the assembly and production of TG-rich VLDL in the liver [19]. The decreased VLDL-TG-secretion ability in steatotic livers of L1-Tg mice might be attributed at least partially to these differences in the mRNA levels that could work synergistically.

## Discussion

**Fig. 5:**
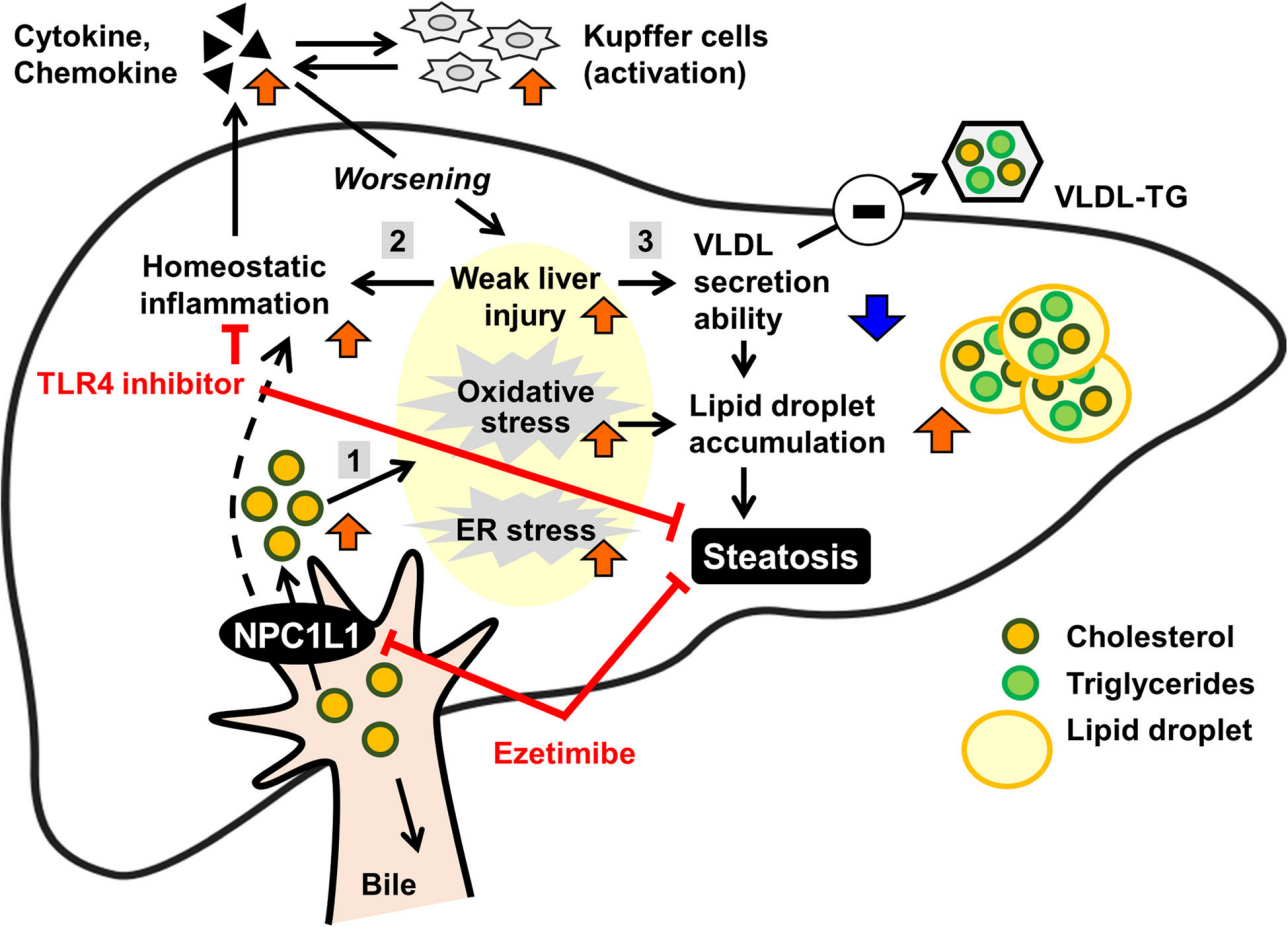
Schematic illustration of the proposed model for hepatic NPC1L1-mediated steatosis induction**.** Hepatic NPC1L1-mediated steatosis can be characterized by at least three features that occur sequentially: (1) involvement of multiple cytotoxic factors that could be trigged by lipid overloading mediated by hepatic NPC1L1 localized on the bile canalicular membrane of hepatocytes; (2) activation of innate immune system and the TLR4-mediated pathway during the early progression of the steatosis; and (3) decreased hepatic ability of very low-density lipoprotein (VLDL)-triglyceride (TG) secretion would contribute to the rapid accumulation of hepatic TG

The present study successfully provided further details regarding the contribution of hepatic NPC1L1 to the exacerbation of steatosis. Moreover, we found that a decrease in VLDL-TG-secretion ability could be involved in hepatic NPC1L1-mediated steatosis (Table 1 and Additional file 1: Figure S5). This decrease was associated with the attenuation of weak liver injury characterized by decreased serum ALT levels (Fig. 3). Although the mechanism by which hepatic NPC1L1 promotes hepatic steatosis remains elusive, given that hepatic NPC1L1 takes up biliary secreted cholesterol into the liver, re-absorbed cholesterol might be a trigger of hepatic stresses that result in hepatic lipid accumulation in certain conditions caused by a hyperlipidemic diet. This interpretation is supported by previous findings that showed oxidative stress, endoplasmic reticulum (ER) stress, and inflammatory cytokines are elevated in steatotic livers of L1-Tg mice fed a HFD [11]. Collectively, findings obtained here provide support for a mechanism underlying the early stage of hepatic NPC1L1-mediated steatosis, which is outlined in Fig. 5.

Results in this study uncovered three features of the early stage of hepatic NPC1L1-mediated steatosis. First, multiple stresses would be involved in its aetiology. Based on the presence of weak liver injury (Fig. 3) as well as the elevation of oxidative and ER stress [11] in the livers of L1-Tg mice fed a HFD, NPC1L1-mediated lipid overload should induce several types of hepatic stresses, resulting in steatosis formation. Indeed, intracellular accumulation of excessive lipid was reported to induce ER stress, which was followed by the acceleration of lipid droplet formation for cytoprotection [20]. Considering the cytotoxicity of excessively accumulated intracellular cholesterol [5, 21], the progression of steatosis in L1-Tg mice, along with the attenuation of weak liver injury, might be part of the intrinsic responses of hepatic protection.

Second, proinflammatory processes could be involved in hepatic NPC1L1-mediated steatosis. LCN2 is a reliable indicator of hepatic damage and positively correlated with inflammation [18]; therefore, the elevation of Lcn2 in the livers of L1-Tg mice fed a HFD (Additional file 1: Figure S3) supports a previous study that shows that the activation of innate immune systems is related to hepatic NPC1L1-mediated steatosis [11]. Indeed, the hepatic NPC1L1-mediated steatosis was prevented by the administration of a toll like receptor 4 (TLR4) inhibitor and attenuated by macrophage depletion. Additionally, given that TLR4 in hepatocytes also plays a pivotal role during the early progression of HFD-induced NAFLD [22, 23] like in non-parenchymal cells, TLR4 activation in hepatocytes could be involved in the induction of steatosis in L1-Tg mice, potentially by synchronizing with hepatic macrophage-mediated inflammatory exacerbation processes (Fig. 5).

The third feature is a rapid decrease in the hepatic VLDL-TG secretion ability (Table 1). Regarding this finding, a previous study provides one supportive observation in which adenovirus-mediated hepatic expression of NPC1L1 in WT mice decreased the VLDL-TG secretion rate [24]. However, this previous study did not report a remarkable accumulation of hepatic TG in the infected mice. We therefore realize that the phenotypic expression of hepatic NPC1L1-mediated steatosis must depend on diet, especially in terms of the quantity and quality of NPC1L1 substrates, such as cholesterol. In this context, dietary components absorbed by intestinal NPC1L1 is also likely to contribute to steatosis formation, because the L1-Tg mice have both hepatic and intestinal expression of NPC1L1, similar to humans. Considering the not-so-limited substrate specificity of NPC1L1 [25–27], some cholesterol derivatives, such as oxidized-cholesterols, might also be involved in the NPC1L1-mediated exacerbation of steatosis.

As an alternative explanation for the notion described above that was discussed from the view point of the role of NPC1L1 as a cholesterol transporter, we may envision another possibility that hepatic NPC1L1 might behave as a receptor for signal or stress inducers in the liver. Although the present study provide no supportive evidence here, this alternative hypothesis regarding the multi- or at least bi-functionality of NPC1L1 is partially supported by previous reports that show a role for NPC1L1 in ezetimibe-sensitive hepatitis C virus cell entry [28] and that NPC1L1-mediated vesicular endocytosis is triggered by substrate binding and inhibited by ezetimibe [29, 30]. Additionally, this study cannot exclude the possibility that the presence of hepatic NPC1L1 in mice may have disturbed the stability of the murine bile canalicular membrane and/or other protein fates. To resolve these concerns, addressing the detailed molecular behavior of NPC1L1 in hepatocytes during the initiation of steatosis must be elucidated in future investigation. Such studies will provide a deeper understanding of the working mechanisms of NPC1L1 and its clinical importance.

Before closing, some limitations warrant mention. As demonstrated, steatosis was induced in mice fed a HFD when the liver expressed NPC1L1 in this study, indicating that this phenotype is governed by hepatic NPC1L1 substantially. On the other hand, given that glucuronide form of ezetimibe undergoes extensive enterohepatic recirculation [31], orally-administered ezetimibe inhibited both intestinal and hepatic NPC1L1. Thus, the current experimental design could not clarify the potential contribution of intestinal Npc1l1 to the steatosis formation in L1-Tg mice, which should be assessed in future studies using an intestinal Npc1l1 specific loss-of-function model. Lastly, detailed molecular components that link hepatic NPC1L1 function and decreased VLDL-TG secretion remain to be elucidated; thus, understanding how re-absorbed biliary cholesterol affect the VLDL-TG formation and/or the secretion process in the liver will be a focus of future research.

## Conclusions

Hepatic NPC1L1 exacerbates diet-induced steatosis, which was accompanied by decreased hepatic ability of VLDL-TG secretion. The results obtained in the present in vivo study provide a better understanding of L1-Tg mice as a promising NAFLD animal model that is able to re-absorb biliary-secreted cholesterol into the liver, similar to humans. Furthermore, this study informs further studies that will address the pathophysiological impact of re-absorbed biliary cholesterol on hepatic lipid homeostasis regulation, including VLDL handling in the liver.

## Additional File

**Additional File 1: Figure S1**. Expression levels of hepatic genes involved in the regulation of biliary cholesterol secretion in WT and L1-Tg mice; **Figure S2**. Photographic images of the livers of L1-Tg mice fed a high-fat diet; **Figure S3**. Expression levels of lipocalin 2 in the livers of WT and L1-Tg mice; **Figure S4**. Expression levels of hepatic genes implicated in the regulation of lipolysis, fatty acid import, and lipogenesis in WT and L1-Tg mice; **Figure S5**. Apparent VLDL-TG-secretion abilities; **Figure S6**. Expression levels of hepatic genes implicated in VLDL assembly and secretion in WT and L1-Tg mice; **Table S1**. Primer sequences for qRT-PCR analysis for each gene in *Mus musculus*; **Table S2**. Serum levels of TG in L1-Tg mice; **Appendix**.

## Data Availability

Data supporting the findings of this study are included in this published article and its Supplementary Information or are available from the corresponding author upon reasonable request.

## Abbreviations

ABC: ATP-binding cassette
B.W.: body weight
CFD: control-fat diet
HFD: high-fat diet
L/B ratio: the ratio of liver weight to body weight
L1-Tg mice: transgenic mice expressing human NPC1L1 in hepatocytes
Lcn2: lipocalin 2
NAFLD: non-alcoholic fatty liver disease
NPC1L1: Niemann-Pick C1-Like 1
TBST: Tris-buffered saline containing 0.05% Tween 20
TG: triglyceride
VLDL: very low-density lipoprotein
WT: wild-type
