# Supplementary material for: Pathophysiological importance of bile cholesterol reabsorption: hepatic NPC1L1-exacerbated steatosis and decreasing VLDL-TG secretion in mice fed a high-fat diet
Source: Lipids Health Dis. 2019 Dec 28;18:234. doi: 10.1186/s12944-019-1179-0 (PMC6935138; doi:10.1186/s12944-019-1179-0)
Supplement: Supplementary file 1 — Additional File 1: Figure S1. Expression levels of hepatic genes involved in the regulation of biliary cholesterol secretion in WT and L1-Tg mice; Figure S2. Photographic images of the livers of L1-Tg mice fed a high-fat diet; Figure S3. Expression levels of lipocalin 2 in the livers of WT and L1-Tg mice; Figure S4. Expression levels of hepatic genes implicated in the regulation of lipolysis, fatty acid import, and lipogenesis in WT and L1-Tg mice; Figure S5. Apparent VLDL-TG-secretion abilities; Figure S6. Expression levels of hepatic genes implicated in VLDL assembly and secretion in WT and L1-Tg mice; Table S1. Primer sequences for qRT-PCR analysis for each gene in Mus musculus; Table S2. Serum levels of TG in L1-Tg mice; Appendix. [file 12944_2019_1179_MOESM1_ESM.pdf]

Supplementary Information

Pathophysiological importance of bile cholesterol reabsorption: hepatic NPC1L1-exacerbated steatosis and decreasing VLDL-TG secretion in mice fed a high-fat diet

Yu Toyoda[1], Tappei Takada[1*], Yoshihide Yamanashi[1], Hiroshi Suzuki[1]

[1]Department of Pharmacy, The University of Tokyo Hospital, Faculty of Medicine, The University of Tokyo, 7-3-1 Hongo, Bunkyo-ku, Tokyo 113-8655, Japan

*** Correspondence:**
Tappei Takada, Ph.D.
tappei-tky@umin.ac.jp

Figure S1

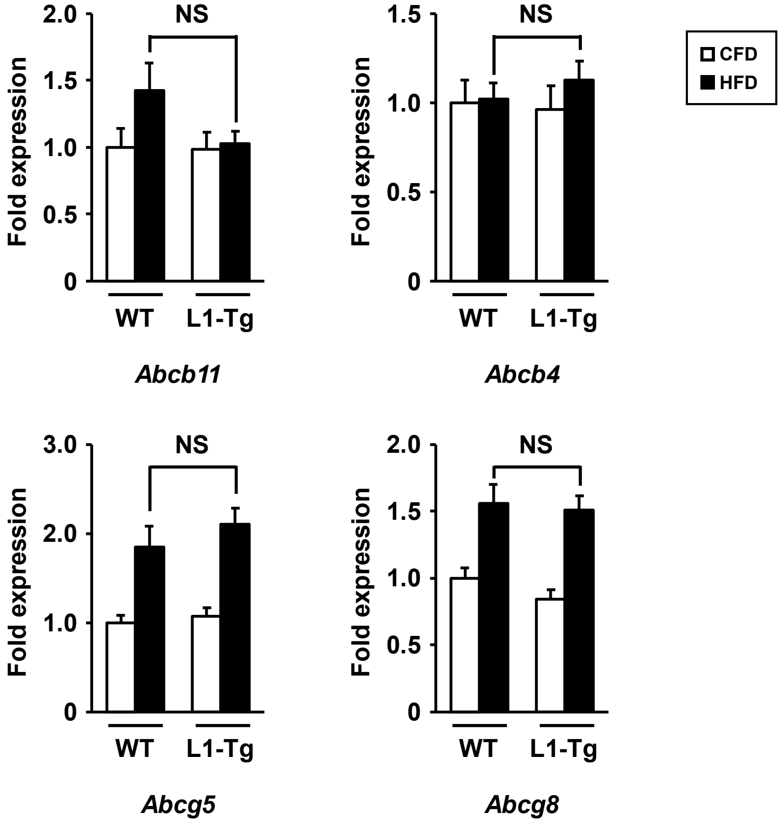

Figure S1. Expression levels of hepatic genes involved in the regulation of biliary cholesterol secretion in WT and L1-Tg mice. To examine the effect of a high-fat diet (HFD) on the expression of each hepatic gene, we analyzed livers from wild-type (WT) and L1-Tg mice fed a control-fat diet (CFD) or HFD for two weeks. In qRT-PCR analyses, *β-actin* mRNA was used as an internal control and fold-changes in the expression levels of each hepatic gene were normalized to the control (WT-CFD) level. Data are expressed as the mean ± SEM. *n* (CFD and HFD) = 9 and 6 (WT); 16 and 15 (L1-Tg). In the four groups, a two-factor factorial ANOVA showed no significant genotype (WT *vs.* L1-Tg) × diet type (CFD *vs.* HFD) interaction and no significant effect of genotype on the fold expression [P = 0.25 (*Abcb11*), 0.82 (*Abcb4*), 0.15 (*Abcg5*), and 0.57 (*Abcg8*)], regardless of diet. On the other hand, with *Abcg5* and *Abcg8*, significant effect of food type on the fold expression ($P < 0.01$) was found. NS, not significantly different between groups (two-sided *t*-test).

Figure S2

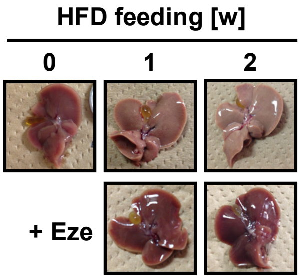

Figure S2. Photographic images of the livers of L1-Tg mice fed a high-fat diet. Administration of ezetimibe (Eze), an NPC1L1-selective inhibitor, completely prevented steatosis formation in L1-Tg mice fed a high-fat diet (HFD). Representative images are shown.

Figure S3

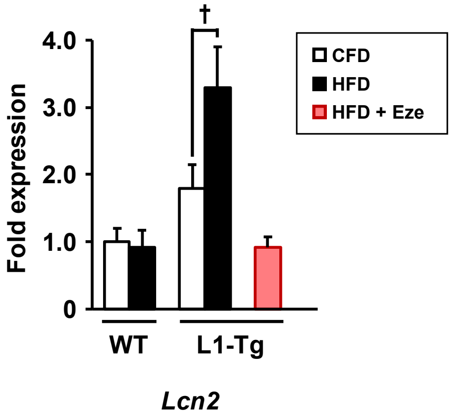

Figure S3. Expression levels of *lipocalin 2* in the livers of WT and L1-Tg mice. To examine the effect of a high-fat diet (HFD) on hepatic expression of the *lipocalin 2* (*Lcn2*) gene, we analyzed livers from wild-type (WT) and L1-Tg mice fed a control-fat diet (CFD) or high-fat diet (HFD) for two weeks. The livers of L1-Tg mice fed a HFD with ezetimibe (Eze) were also analyzed. In qRT-PCR analysis, *β-actin* mRNA was used as an internal control and fold-changes in the expression levels of hepatic *Lcn2* were normalized to the control (WT-CFD) level. Data are expressed as the mean ± SEM. *n* (CFD and HFD) = 9 and 5 (WT); 16 and 15 (L1-Tg); 8 (L1-Tg-HFD with Eze). In the four groups that were not administered Eze, a two-factor factorial ANOVA showed no significant genotype (WT *vs.* L1-Tg) × diet type (CFD *vs.* HFD) interaction and significant effects of genotype on the fold expression (P = 0.011). Statistical analyses for significant differences between groups were performed using a two-sided t-test (†, $P < 0.05$).

Figure S4

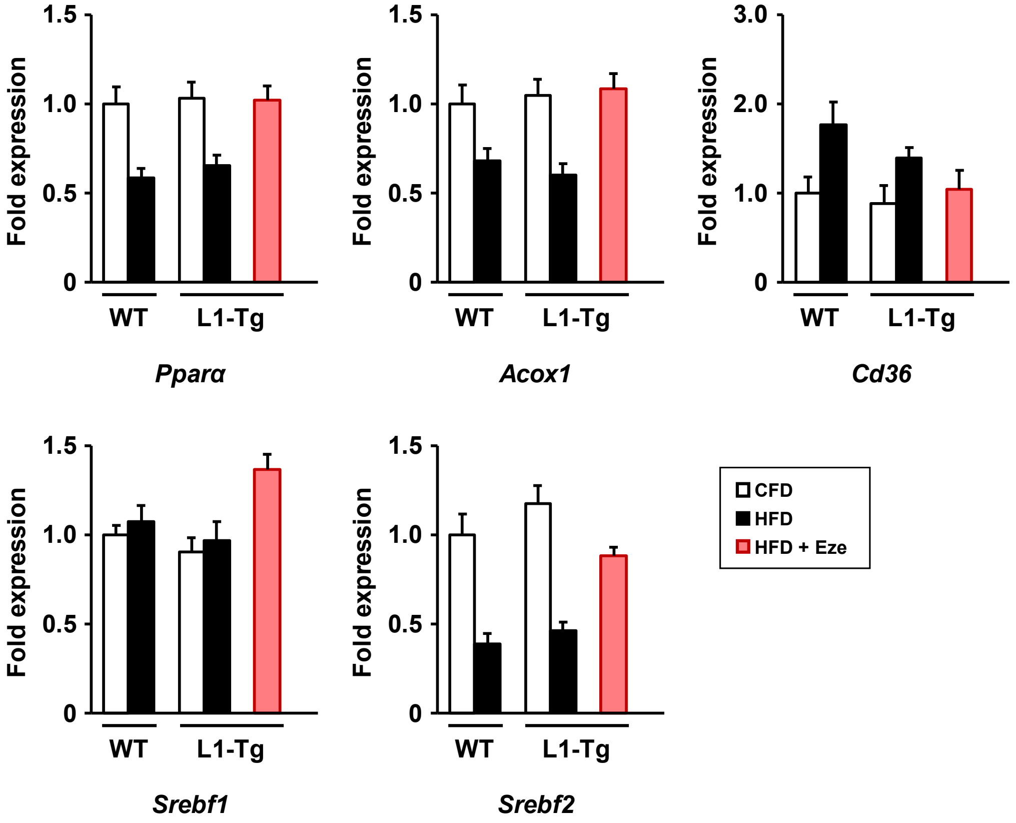

Figure S4. Expression levels of hepatic genes implicated in the regulation of lipolysis, fatty acid import, and lipogenesis in WT and L1-Tg mice. To examine the effect of a high-fat diet (HFD) on the expression of each hepatic gene, we analyzed livers from wild-type (WT) and L1-Tg mice fed a control-fat diet (CFD) or high-fat diet (HFD) for two weeks. The livers of L1-Tg mice fed a HFD with ezetimibe (Eze) were also analyzed. In qRT-PCR analyses, *β-actin* mRNA was used as an internal control and fold-changes in expression levels of each hepatic gene were normalized to the control (WT-CFD) level. Data are expressed as the mean ± SEM. *n* (CFD and HFD) = 9 and 6 (WT); 16 and 15 (L1-Tg); 8 (L1-Tg-HFD with Eze). In the four groups that were not administered Eze, a two-factor factorial ANOVA showed no significant genotype (WT *vs.* L1-Tg) × diet type (CFD *vs.* HFD) interaction and no significant effect of genotype on the fold expression [P = 0.60 (*Pparα*), 0.95 (*Acox1*), 0.26 (*Cd36*), 0.33 (*Srebf1*), and 0.19 (*Srebf2*)], regardless of diet.

Figure S5

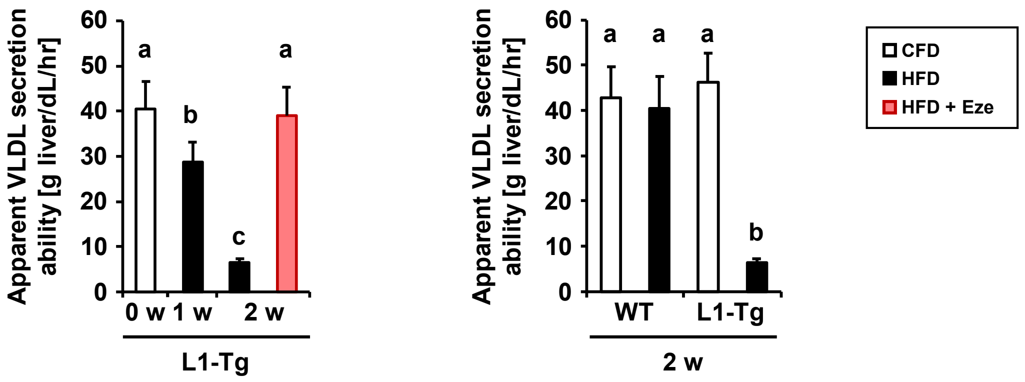

Figure S5. Apparent VLDL-TG-secretion abilities. Wild-type (WT) and L1-Tg mice were fed a control-fat diet (CFD) or high-fat diet (HFD) with or without ezetimibe (Eze) for indicated periods, then subjected to the determination of apparent very low-density lipoprotein-triglyceride (VLDL-TG)-secretion ability, which was calculated by dividing the VLDL-TG-secretion rate [mg of TG/dL of serum/hr] by the average hepatic TG levels [mg of TG/g of liver]. Data are expressed as the mean ± SEM. The *left* panel represents the information of **Table 1** as a bar chart; n = 4 [0 w]; 7 [1 w]; 7 (HFD), 4 (HFD + Eze) [2 w]. In the *right* panel, n (CFD and HFD) = 4 and 7 (WT); 6 and 7 (L1-Tg). Statistical analyses for significant differences were performed using Bartlett's test, followed by a non-parametric Steel–Dwass test for multiple comparisons. Different letters indicate significant differences between groups ($P < 0.05$) in each panel. w, weeks.

Figure S6

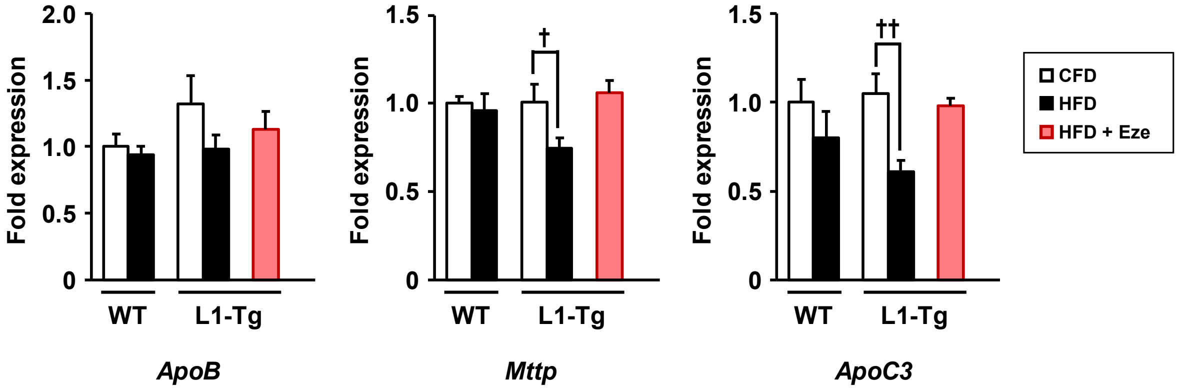

Figure S6. Expression levels of hepatic genes implicated in VLDL assembly and secretion in WT and L1-Tg mice. To examine the effect of a high-fat diet (HFD) on the expression of each hepatic gene, we analyzed the livers of wild-type (WT) and L1-Tg mice fed a control-fat diet (CFD) or high-fat diet (HFD) for two weeks. The livers of L1-Tg mice fed a HFD with ezetimibe (Eze) were also analyzed. In qRT-PCR analyses, *β-actin* mRNA was used as an internal control and fold-changes in the expression levels of each hepatic gene were normalized to the control (WT-CFD) level. Data are expressed as the mean ± SEM. *n* (CFD and HFD) = 9 and 6 (WT); 16 and 15 (L1-Tg); 8 (L1-Tg-HFD with Eze). Statistical analyses for significant differences were performed using a two-sided *t*-test (†, $P < 0.05$; ††, $P < 0.01$).

Table S1

Table S1. Primer sequences for qRT-PCR analysis for each gene in *Mus musculus*.

Symbol	Gene name		Sequence 5′ to 3′
β-Actin	Actin, beta	F<br>R	AGATCAAGATCATTGCTCCTCCTG<br>AACGCAGCTCAGTAACAGTCC
Abcb4	ATP-binding cassette, sub-family B, member 4	F<br>R	ACTAGGCAGCATCAGCAACC<br>GAGCTATGGCCATGAGGGTG
Abcb11	ATP-binding cassette, sub-family B, member 11	F<br>R	TCACATCTGTAGGGTTGTTGAG<br>CAATGCGCACACACTTCCC
Abcg5	ATP-binding cassette, sub-family G, member 5	F<br>R	CCTGCAGAGCGACGTTTTTC<br>CCAATCATTTGGTCCGCCAC
Abcg8	ATP-binding cassette, sub-family G, member 8	F<br>R	CTTCAGGATGCTTCGCAGGG<br>GGCTGCTATGAGACCTCCAG
Acox1	Acyl-Coenzyme A oxidase 1, palmitoyl	F<br>R	ATCCTGAGCCTTTGGACCTTC<br>TCGAAGATGAGTTCCATGACCC
ApoB	Apolipoprotein B	F<br>R	AAACATGCAGAGCTACTTTGGAG<br>TTTAGGATCACTTCCTGGTCAAA
ApoC3	Apolipoprotein C-III	F<br>R	GCATCTGCCCGAGCTGAAGAG<br>CTGAAGTGATTGTCCATCCAGC
Cd36	CD36 molecule	F<br>R	GTGCAGTCCTGGCTGTGTTTG<br>CAGACAGTGAAGGCTCAAAGATGG
Lcn2	Lipocalin 2	F<br>R	CGGACTACAACCAGTTCGCC<br>ACGTTCCTTCAGTTCAGGGG
Mttp	Microsomal triglyceride transfer protein	F<br>R	CTCTGCTTCCGTTAAAGGTCAC<br>GAGATTTTGTAGCCCACGCTG
Pparα	Peroxisome proliferator activated receptor alpha	F<br>R	CGCTATGAAGTTCAATGCCTT<br>TGCAACTTCTCAATGTAGCC
Srebf1	Sterol regulatory element binding transcription factor 1	F<br>R	GGAACTTTTCCTTAACGTGGGC<br>TGAGCTGGAGCATGTCTTCG
Srebf2	Sterol regulatory element binding transcription factor 2	F<br>R	TGGAAGTGACCGAGAGTCCC<br>GAGACTGCTCCACAGGTGAC

F, forward; R, reverse.

Table S2

Table S2. Serum levels of TG in L1-Tg mice.

Diet type	Ezetimibe	Feeding time	Serum TG [mg/dL]	*n*
CFD	−	1 w	76.3 ± 7.0	7
	−	2 w	69.5 ± 4.4	10
HFD	−	1 w	91.4 ± 6.8[a]	7
	−	2 w	53.4 ± 5.9[b]	8
	+	2 w	90.8 ± 11.4[a]	4

Values are expressed as the mean ± SEM. Statistical analyses of significant differences among the groups in each diet type were performed using a two-sided *t*-test (two CFD groups) or Bartlett's test, followed by a parametric Tukey–Kramer multiple-comparison test (three HFD groups). Different letters indicate significant differences between groups ($P < 0.05$). CFD, control-fat diet; HFD, high-fat diet; w, weeks; TG, triglyceride.

Appendix

Appendix 1: The values of mean and SEM in Figure 2b.

Feeding time	Hepatic cholesterol [mg/g liver] (X axis)	Hepatic TG [mg/g liver] (Y axis)
0 w	5.83 ± 0.51	11.51 ± 1.45
1 w	13.06 ± 1.32	17.98 ± 2.28
2 w	20.00 ± 1.12	22.72 ± 1.34
3 w	24.89 ± 0.82	51.09 ± 3.33
6 w	39.93 ± 2.60	51.40 ± 4.02

Appendix 2: The values of mean and SEM in Figure 2c.

Feeding time	L/B ratio [%]
0 w	4.80 ± 0.22
1 w	6.13 ± 0.15
2 w	7.02 ± 0.35
3 w	7.75 ± 0.18
6 w	9.62 ± 0.82
